# Muscular Strength, Power, and Endurance Adaptations after Two Different University Fitness Classes

**DOI:** 10.3390/sports9080107

**Published:** 2021-07-28

**Authors:** Brittany S. Hollerbach, Sarah J. Cosgrove, Justin A. DeBlauw, Nattinee Jitnarin, Walker S. C. Poston, Katie M. Heinrich

**Affiliations:** 1 Center for Fire, Rescue and EMS Health Research, NDRI-USA, Inc., 1920 W 143rd Street, Suite 120, Leawood, KS 66224, USA; jitnarin@ndri-usa.org (N.J.); poston@ndri-usa.org (W.S.C.P.); 2 Department of Kinesiology, Kansas State University, Natatorium 8, 920 Denison Ave, Manhattan, KS 66506, USA; sarahjoann17@gmail.com (S.J.C.); jdeblauw@ksu.edu (J.A.D.)

**Keywords:** weight training, resistance training, physical education, CrossFit, high-intensity functional training, college students, physical activity, body weight

## Abstract

Physical activity (PA) classes help college students add weekly PA, which can help improve health and maintain body weight. Traditional weight training (TWT) can improve strength and aerobic capacity. High-intensity functional training such as CrossFit^®^ (CF) provides time-efficient workouts with both muscle strengthening and aerobic exercises. Limited research has compared these classes for college students. We examined changes in muscular strength, power, and endurance as well as body composition. Participants were 85 healthy college students enrolled in TWT (*n* = 36, age 22.6 ± 4.1 years, 72.2% male) or CF (*n* = 49, age 21.8 ± 3.2 years, 55.1% male) classes meeting twice/wk for 8 weeks between October 2017 and May 2018. Baseline and posttest measurements included a vertical jump, grip strength, a 2 min push-up test, a 1 min squat test, height, weight, and a bioelectrical impedance analysis. Although no significant group × time interactions were found, there was a significant main effect of time for push-ups and squats (both *p* < 0.001). Participants enjoyed the classes and most planned to continue. Both classes improved muscular endurance although no significant differences were found between them. Activity classes provide college students with an option for increasing their weekly PA and help maintain body composition. Future research should examine the benefits from longer or more frequent classes.

## 1. Introduction

The United States Department of Health and Human Services (USDHHS) recommends individuals engage weekly in moderate to vigorous aerobic physical activity (PA) as well as muscle strengthening activities [1]. People who are physically active tend to live longer and have a lower risk for heart disease, a stroke, type 2 diabetes, depression, and even a few cancers [1]. PA can aid in maintaining body weight and helps improve cognition across the lifespan [1]. Although most universities offer a variety of PA options, nearly a quarter (22.4%) of students do not engage in regular PA and gain significantly more weight than their age-matched peers that do not attend college or university [2,3,4]. Examining PA classes designed for college students is relevant and may assist in determining the best types of classes to offer to improve health, prevent weight gain, and increase adherence in college students.

Nationally, more than two thirds (70.7%) of U.S. adults are overweight or obese and obesity has increased dramatically among young adults aged 18–29 [5,6]. Recent data from the American College Health Association (ACHA) indicate 37.7% of college students are overweight or obese [7]. The transition to college includes many social forces that can negatively impact students’ nutrition, drinking, and PA habits [2,8]. This is reflected by a period of weight gain during the freshman year where the majority of students (60.9%) gain an average of 3.38 kg [3]. Even more concerning is that weight gain significantly increases across students’ time in college [9] and weight trajectories during adolescence persist into adulthood [10].

Enrolling in activity classes such as weight training helps college students add scheduled PA into their week. The benefits of traditional weight training (TWT), or the practice of movement under load with a weighted object, are widely understood and accepted by a variety of health professionals [11] and include improvements in muscular strength, power, hypertrophy, and muscular endurance as well as neuromuscular adaptations [12]. TWT is a popular form of exercise recommended for many different populations that has been widely examined in the literature and is commonly offered as an activity class for college students [13,14].

High-intensity functional training (HIFT) exercise programs have grown in popularity in recent years but remain relatively understudied [15]. CrossFit^®^ (CF), a form of high-intensity functional training (HIFT), emphasizes functional, multijoint movements via both aerobic and muscle strengthening exercises that improve multiple fitness domains [15,16]. CF adds competition to the attainment of multidimensional fitness, which may help explain its rise in popularity [16,17,18]. CF workouts attempt to maximize the amount of work done in the shortest amount of time, making them a time-efficient, high-intensity exercise option that can improve aerobic fitness as well as muscular strength [15]. In addition, improvements in body composition have occurred after as few as 15 sessions [19]. This time-efficient option might benefit an already time-constrained college population [20] and can be offered as an activity class option.

The current literature supports that CF has a high aerobic stimulus due to minimal rest periods and has been shown to increase maximal aerobic capacity (VO2max) [21,22]. Limited research exists comparing muscular adaptations in CF to those in TWT. One study examined the effects of CF compared to TWT in a college population enrolled in 16-week courses [22]. Participants were matched on baseline push-up and vertical jump performances. The CF group improved aerobic capacity and had greater increases (22%) in muscular endurance than the TWT group (0%) [22]. Though an excellent first step, this study did not examine multiple muscular adaptations as the only measures of muscular fitness were push-ups and vertical jumps. Our study builds on this initial investigation by additionally examining grip strength and squats as well as body composition. The purpose of the present investigation was to examine the differences in muscular strength, power, and endurance between two distinct college fitness classes: Introduction to CF and TWT. We hypothesized that the CF group would see the same if not better muscular strength adaptations than the TWT group. Changes in body composition were examined as a secondary outcome.

## 2. Materials and Methods

### 2.1. Study Design

This observational cohort study examined four different cohorts of college students between October 2017 and May 2018 at a large Midwestern university. Cohorts were enrolled over seven months and were registered for 1 credit hour in 8-week courses during the fall and spring semesters. Participants were offered an incentive of a jump rope valued at USD 10, a t-shirt, or a USD 10 gift card for their participation.

### 2.2. Participants

**Figure 1:**
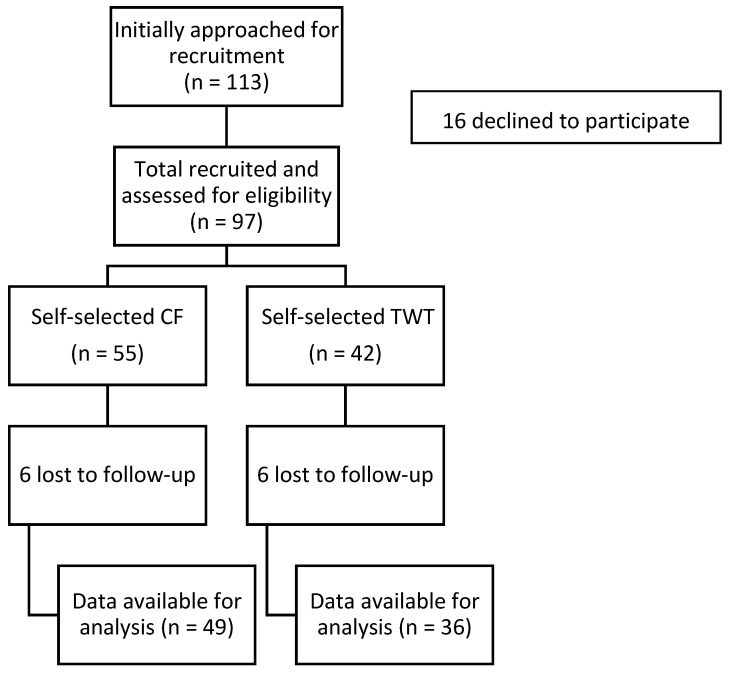
Participant flow through the study.

One hundred and thirteen students enrolled in two different types of intact 8-week fitness courses (CF and TWT) were recruited and screened for eligibility of which 97 (86%) agreed to participate and completed the written informed consent. The inclusion criteria were: a current student enrolled in CF or TWT classes and to complete the Physical Activity Readiness Questionnaire (PAR-Q) by answering “no” to all questions [23]. The participant flow is illustrated in Figure 1.

The exclusion criteria were being under the age of 18 years or having any known cardiovascular, metabolic, or respiratory disease. Having an internal pacemaker would preclude the participant from completing the Tanita body composition assessment although no participants reported having one. There was no coercion as participants could partake in either class without being a part of the research study and could discontinue study participation at any time. The study was approved by the Kansas State University Institutional Review Board (IRB#8885).

Of the 113 students initially approached, 97 consented to participate (CF mean age: 21.7 ± 3.0 years, 54.5% male; TWT: 22.3 ± 3.9 years, 69.0% male). All 113 students completed the fitness measures during class; however, data were only recorded for the 97 students who agreed to participate in the study. Twelve participants (CF = 6, TWT = 6) who initially enrolled in the study dropped their respective class (CF or TWT) and did not complete the follow-up testing session and were considered “lost to follow-up.” Participants completed baseline and follow-up assessments, which occurred during their first and last class sessions, to determine the changes over 8 weeks.

### 2.3. Measures

Baseline and follow-up questionnaires assessed basic demographics including age, sex, and race. Race was dichotomized into “Minority” (Black/African American; American Indian/Alaskan Native; Asian; and Native Hawaiian/Other Pacific Islander) and “Non-Minority” (White/Caucasian). Muscular power was assessed by a countermovement vertical jump (VJ) using Vertec (Jump, Sunnyvale, CA) where participants were allowed to do a countermovement with the lower limbs before jumping [24]. The VJ has excellent reliability (α = 0.98) and factorial validity (λ = 0.87) [24]. Upper body muscular strength (grip strength; GS) was assessed with the Takei 5401 Hand Grip Dynamometer (Digital; Niigata City, Japan). Research has shown that a hand grip dynamometer is a valid and reliable measure (*p* < 0.05) and is a strong correlate (r > 0.9994) with upper body muscular strength [25,26]. The dominant hand of participants was noted; grip strength (GS) was recorded three times for both hands in an alternating fashion. The best of three attempts was recorded for each hand.

Muscular endurance was assessed with a 2 min timed push-up (PU) test (upper body muscular endurance) [27] and a 1 min body weight squat (SQ) test (lower body muscular endurance) [28]. The PU test required participants touch their chin to the mat and the score was the number of continuous repetitions completed [27]. Men did the push-ups from their toes; women did a modified push-up from their knees [27]. The SQ test required participants to lower themselves until their buttocks touched a 14 inch (diameter) Dynamax medicine ball and then return to the standing position (full extension of the hips and knees); the score was the number of repetitions completed in one minute. The SQ test has been found to have good test-retest reliability (0.4% coefficient of variance) [29].

A Seca stadiometer (Chino, CA, USA) was used to assess height. The Tanita TBF-300A digital bioelectrical impedance analysis (BIA) scale (Arlington Heights, IL, USA) was used to assess fat mass (FM), fat free or lean mass (FFM), percent body fat (BF%), body mass index (BMI), and weight (to the nearest 0.1 kg). Research has shown that BIA correlates well (r > 0.8) with the gold standard measure of a dual x-ray absorptiometry (DEXA) analysis for body composition [30].

Class feedback surveys were completed at the end of TWT and CF classes and asked the participants qualitatively about their experience on their respective course and if they planned to continue that type of exercise. A thematic qualitative analysis was conducted by two researchers for responses to the questions.

### 2.4. Activity Classes

Participants were recruited from intact PA classes (TWT or CF) at a Midwestern university. All fitness levels and abilities were able to enroll. Physical assessments were conducted during the first week (baseline) and the final week (follow-up) of the 8-week courses. The lead researcher trained all data collectors on examination procedures to ensure that all participants were evaluated by the same raters and under the same conditions for both baseline and follow-up assessments.

Both courses met twice weekly for 8 weeks. Students were asked to log their workouts (both in class and additional workouts) either in a paper journal or electronically. Course instructors gathered this material at the end of the 8-week courses and copies were provided to the researchers with the participants’ consent along with attendance records. While class frequency was matched between classes, training volume was not.

#### 2.4.1. Introduction to CrossFit (CF)

The Introduction to CF class was designed to introduce the students to CF workouts and programming methodology including functional movements with emphasis on maintaining proper posture through the body’s normal range of motion and how to safely increase intensity. The exercise was designed to be completed at a high intensity relative to each student’s respective fitness level. The class focused on technique and moving between positions in a safe and efficient manner. Basic bodyweight exercises were utilized to build general physical skills. The curriculum continued to increase intensity by increasing load or decreasing time (completing work faster). In general, class sessions included a general warm-up of 10–15 min, a brief introduction to the workout of the day and the movements included in it, 10–20 min of practicing the movements, a 10–25 min workout of the day, and a 5–10 min cool down. Examples of class workouts are listed in Table 1. Note that the loads used were individually determined in consultation with the course instructor, a CF Level 4 Coach. Classes were 75 min in length.

#### 2.4.2. Traditional Weight Training (TWT)

Strength Training for Fitness and Introduction to Weightlifting exercise classes were examined as TWT programs. Classes were 60 min in duration and were taught by a graduate student in kinesiology with at least a bachelor’s degree in kinesiology. The TWT courses utilized an undulating periodization training program developing multiple fitness characteristics in beginner-level to novice-level exercisers. Each class started with a standardized warm-up followed by a 5–10 min review of the day’s training, then approximately 30 min of exercise. The course utilized body weight, a barbell, free weights, and machine training and modulated the intensity on an individual basis. The intensity/load increased throughout the course. Examples of the workouts are listed in Table 2. A “complex” is a series of movements performed back-to-back in which the set number of repetitions is completed for each movement before moving on to the next. Tempo training is a technique used to develop time under tension or focus on a particular area of a movement pattern.

### 2.5. Statistical Approach

IBM SPSS version 25 (Armonk, NY, USA) was used for the data analysis. Variables are presented as means and standard deviations or percentages. Statistical significance was set at *p* < 0.05. We examined baseline differences using independent sample *t*-tests both for completers versus non-completers within each group as well as for completers between groups. A chi-squared analysis was conducted for the categorical variables. After examining the data for normality, a two-way analysis of variance (ANOVA) with repeated measures was conducted to determine the effect of class participation on each fitness and body composition variable. The group (CF versus TWT) was used as a between-subject factor and time (baseline versus posttest) was used as a within-subject factor. To control for Type 1 errors, the alpha level was adjusted using the Bonferroni correction. Additionally, program feedback was examined qualitatively and common responses were noted.

## 3. Results

Baseline participant characteristics are provided in Table 3. At the baseline, there were no statistically significant differences between the groups. However, we did find significant differences between dropouts and completers only for the CF class participants. Specifically, there was a significant difference in baseline BF%, *t* = 2.871, *p* = 0.006, where those who dropped out had significantly greater BF% at baseline (37.2 ± 6.9% versus 24.8 ± 9.4%); and in baseline squat repetitions, *t* = 2.536, *p* = 0.014, where those who dropped out completed fewer squats at the baseline (30.4 ± 17.3 versus 41.7 ± 8.5 repetitions).

CF class attendance was reported for all 49 participants and had a mean attendance rate of 88.6 ± 11.1%. TWT class attendance was reported for 30 of 36 participants with a mean attendance rate of 88.0 ± 12.0%. An independent sample *t*-test found that attendance did not significantly differ by class type (*t* = 0.19, *p* = 0.85). No participants reported any injuries from class participation that impacted on their training.

**Figure 2:**
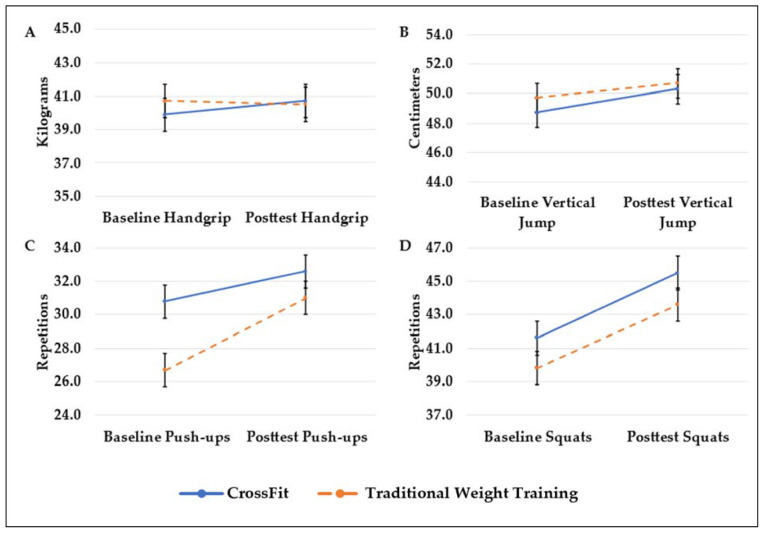
(**A**–**D**) Changes in fitness tests over time by group.

**Figure 3:**
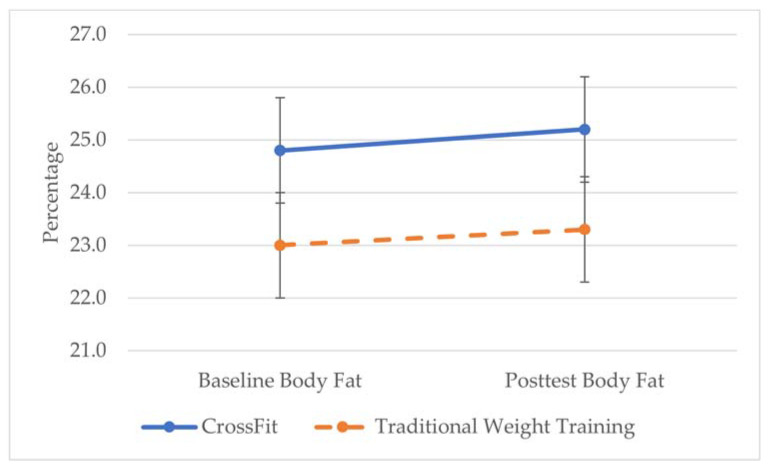
Changes in body fat percentage (BF%) over time by group.

Changes in fitness and body composition (M ± SD) after 8 weeks by class type are shown in Figure 2A–D and Figure 3. There was no significant group × time interaction for any of the measures. However, there was a significant main effect of time for push-ups (ƒ = 16.12, *p* < 0.001, η^2^ = 0.032) and squats (ƒ = 40.87, *p* < 0.001, η^2^ = 0.333) with both groups improving over 8 weeks.

Overall, participants in both TWT and CF reported positive experiences in their respective classes. Participants enjoyed the CF workouts and noted the variety and liked how short the workouts were. They also indicated that the workouts were intense yet attainable; “fun and challenging”. The majority of respondents (67%) planned to continue taking CF; 27% said they might and only 6% said they would not.

The TWT participants enjoyed the flexibility of their workouts and the fundamentals learned in the class. Respondents noted that the progression of the class was a bit slow and a few workouts were “easy” but they reported that they learned a lot. Many respondents discussed the benefit of learning the fundamentals of weightlifting and they enjoyed that type of introduction. The majority of respondents (77%) planned to continue taking TWT; 10% said they might and 13% reported they would not be interested in continuing that type of training in the future.

## 4. Discussion

The purpose of the present investigation was to examine muscular strength and endurance adaptations and changes in body composition in college students enrolled in two different college fitness classes, CF and TWT. Our hypothesis that the CF group would see the same if not better muscular strength and endurance adaptations than the TWT group was partially supported as there was a significant main effect for time for both groups in muscular endurance (i.e., increased push-up and squat repetitions). However, these improvements were not significantly different between groups. In addition, participants in both groups maintained body composition, an important note in a population where weight gain is prevalent.

Few studies to date have examined muscular endurance and strength adaptations from CF compared to a more traditional weightlifting program and only one has done so among college students [22]. A recent systematic review summarized how CF interventions have improved strength, power, muscular endurance, and body composition among other factors [31]. However, similar to our study, research with teens found no significant improvements in grip strength after CF participation although the same study also found no improvements in push-ups [32]. While both groups improved push-ups in our study, a previous examination of college students found a 22% increase in push-ups for CF class participants but a 0% increase in push-ups for TWT class participants [22]. That study [22] was 14 weeks in length instead of 8 weeks, which may account for the greater percent increase in push-up performance. Although our sample size was larger, we saw no significant differences between the two types of exercise classes after 8 weeks, suggesting longer (greater than 8-week) courses may be necessary to observe these adaptations in aggregate. Of note, aggregate data miss the heterogeneity of individual results. Future studies might examine each individual’s changes over time as a few individuals may have been highly respondent to the stimulus while others were not. Further, it can be difficult to compare studies due to differences in programming. Barfield and Anderson [22] required participants to travel off-campus to a CrossFit gym where researchers had less control over programming. Our study was completed in a university-based gym where researchers could control the programming and observe participant adherence.

Our courses were only 8 weeks in length whereas similar studies were 14–16 weeks in length [22,33]. The duration of the CF classes in this study was also 15 min longer than the TWT classes although participants spent similar amounts of time completing each day’s workout. A larger dose of PA (either longer class periods, meeting more than twice per week, or meeting for longer than 8 weeks) might have resulted in more statistically significant changes. Additionally, age, sex, and training status play a role in muscular adaptations [34]. Future investigations of this population with a larger sample size will allow investigators to examine additional comparisons including stratification by age and sex.

There are several strengths in our study. First, we were able to recruit a large number of students to participate, making the sample relatively diverse and more generalizable to the college population as a whole. Additionally, we examined multiple measures of muscular adaptation and the coaches delivering the training had expertise in these topics. We also collected attendance records for each class. Future research should examine the feasibility of encouraging college student participation in fitness courses as a way of increasing time spent in PA.

Our primary limitation was not being able to randomize participants into conditions. Another limitation was not being able to match participants based on training volume. Similar studies have echoed that a valid method of matching work output must be developed to make empirical comparisons between traditional and non-traditional programs. As all assessments were conducted within the class time (i.e., 60 and 75 min), other limitations were equipment availability and time for assessments. We used general health-related physical fitness variables; however, there may be a need for more exercise type-specific tests to detect differences. In particular, the assessment of changes in aerobic capacity as well as an additional examination of both dominant and non-dominant hand grip strength may be beneficial for CF participants.

We observed 8-week sessions across two semesters; therefore, there were possibly seasonal effects due to collecting data from the beginning, middle, and end of the fall and spring semesters as is consistent with the literature [35]. This may have impacted participants’ motivation and fitness goals, not to mention their other physical activities outside of the classes. Further, we did not objectively measure the participants’ total PA or nutritional habits, which could impact their fitness improvements and body composition. Lastly, our sample was too small to effectively make comparisons by sex, which is important when examining differences in muscular adaptations [34].

## 5. Conclusions

While there are multiple, diverse PA class offerings at universities, CF is one that is popular yet still relatively unexplored in the scientific literature. This study provides evidence of changes in muscular endurance associated with two different types of college PA classes, CF and TWT. Participants also maintained body composition over 8 weeks. This study provides alternative options for exercise prescription designed for improving health, body composition, and muscular endurance for college students. Further investigation is necessary to examine PA classes of a longer duration as well as the efficacy of different types of HIFT programs among the college student population. Furthermore, research should examine if time spent in college PA classes helps students meet PA guidelines.

## Figures and Tables

**Table 1:** Example workouts from the CrossFit class.

Example Workouts	Exercises
5 Rounds for Time	6 Wallballs
	3 Burpees
3 Rounds for Time	3 Barbell Presses
	8 Barbell Jerks
	20 Mountain Climbers (Each Side)
For Time	20 Barbell Front Squats
	20 Barbell Hang Power Cleans
	20 Barbell Thrusters

**Table 2:** Example workouts from the traditional weight training (TWT) classes.

Example Workouts	Exercises	Reps	Rest	Sets
Barbell/Dumbbell Complex	8 Deadlifts			
	8 Bent-Over Rows			
	8 Upright Rows			
	8 Thrusters			
	8 Overhead Squats			
Tempo Training (2-0-2-0)	Squat	10	60 s	3
	Bench Press			
	Push-p			

**Table 3:** Baseline participant characteristics by type of class.

Characteristic	CrossFit (*n* = 49)	Traditional Weight Training (*n* = 36)	*p*-Value
Age (Years)	21.8 ± 3.2	22.6 ± 4.1	0.308
Men (%)	55.1	72.2	0.107
Race (%)			0.199
*Non-Minority*	79.6	72.2	
*Minority*	18.3	27.8	
*Did Not Report*	2.0		
Grip Strength (kg) ^1^	39.9 ± 11.0	40.7 ± 10.3	0.742
Vertical Jump (cm)	48.7 ± 12.0	49.7 ± 12.8	0.948
Push-Ups (Repetitions)	30.8 ± 13.2	26.0 ± 11.2	0.085
Squats (Repetitions)	41.7 ± 8.5	39.8 ± 8.0	0.312
Body Fat (%)	24.8 ± 9.4	22.8 ± 9.2	0.337

^1^ Grip strength is reported for the dominant hand.

## Data Availability

Data are available by request from the corresponding authors.
